# The role of proteinuria on a simple urinalysis in neonatal hypoxic–ischemic encephalopathy: association with clinical and neurodevelopmental outcomes

**DOI:** 10.3389/fneur.2025.1451346

**Published:** 2025-03-18

**Authors:** Ya-Chun Hu, Ji-Nan Sheu, Inn-Chi Lee

**Affiliations:** ^1^Department of Pediatrics, Chung Shan Medical University Hospital, Taichung, Taiwan; ^2^Division of Pediatric Neurology, Department of Pediatrics, Chung Shan Medical University Hospital, Taichung, Taiwan; ^3^Institute of Medicine, School of Medicine, Chung Shan Medical University, Taichung, Taiwan; ^4^Division of Pediatric Nephrology, Department of Pediatrics, Chung Shan Medical University Hospital, Taichung, Taiwan

**Keywords:** newborn, hypoxic–ischemic encephalopathy, biomarker, proteinuria, neurodevelopmental outcomes

## Abstract

**Background:**

Early diagnosis and initiation of hypothermia therapy for neonatal hypoxic–ischemic encephalopathy (HIE) are critical within the first 6 h after birth. Collecting urine, especially from neonates with HIE, can be challenging and time-consuming post-birth due to the likelihood of renal injury.

**Purpose:**

We assessed whether urine protein levels, measured via a simple urinalysis on the first day, could correlate with the outcomes of neonatal HIE.

**Methods:**

We conducted urine analyses of patients with neonatal HIE on the first day to establish a correlation between the severity of HIE and neurodevelopmental outcomes at ≥1 year of age. Eighty-three patients were enrolled, encompassing cases of mild (*n* = 37), moderate (*n* = 30), and severe (*n* = 16) HIE. Three cases were excluded due to mortality caused by severe HIE with associated auria. Based on urine protein levels, patients were grouped as 0 to 30 mg/dL (group 1), 30 to 100 mg/dL (group 2), 100 to 300 mg/dL (group 3), and ≥ 300 mg (group 4).

**Results:**

Urine protein levels were correlated with serum lactic acid levels [*p* = 0.006; *r* (81) = 0.304; *n* = 83], clinical staging [*p* = 0.001; *r* (81) = 0.36], and neurodevelopmental outcomes at ≥1 year of age [*X*^2^ (3, *n* = 83) = 11.35; *p* = 0.009]. The odds ratio for moderate-to-severe HIE in group 4 patients was 7.66 [*p* = 0.010; 95% *confidence interval* (CI), 1.61–36.33] compared with those in groups 1–3. Those in group 4 had a high positive predictive value (87.50%) and high specificity (94.59%).

**Conclusion:**

Elevated urine protein levels observed in the first urinalysis conducted on the day after birth were found to be associated with serum lactic acid levels, clinical staging, and neurodevelopmental outcomes at ≥1 year of age.

## 1 Introduction

Neonatal hypoxic–ischemic encephalopathy (HIE) is a physiological disorder that is caused by a prolonged or profound mismatch between oxygen demand and oxygen delivery in newborn infants (1). Neonatal HIE is a major cause of neonatal mortality, accounting for about 24% of neonatal deaths (2–4). Neonatal HIE can range from mild to severe. When a neonate has moderate to severe HIE, irreversible cerebral cell damage and death can occur, leading to a syndrome of HIE. HIE can affect multiple organs, including the brain, heart, liver, hemopoietic system, and kidney, and may lead to an altered conscious state, autonomic instability, seizures, decreased cardiac output, liver and renal dysfunction, and even death. HIE is thus an important and major cause of neonatal death and neurodevelopmental consequences (5, 6).

Rescue hypothermia is a treatment for neonatal HIE that has been shown to be effective and associated with few adverse effects in newborns (7–9). While therapeutic hypothermia is used clinically to reduce neurological injury secondary to HIE, it is associated with a 45–55% risk of moderate–severe disability or death (7, 8, 10). Therapeutic hypothermia can be performed as a standard of care for moderate-to-severe HIE; however, in newborns with mild HIE, the benefits have not been confirmed and potential consequences include coagulation disorders and cardiopulmonary instability (11, 12). Therapeutic hypothermia has also been shown to improve the function of other organs, such as the kidneys. The presence of shock, low blood pressure and cardiac output, or hypoxic injury to the kidney may indicate renal injury secondary to neonatal HIE, which may further aggravate the morbidity and mortality associated with HIE.

Neonatal HIE can cause multiple organ injury to the brain, heart, liver, and kidneys, among others. While about 34% of cases of HIE are only result in mild organ injury not evident by clinical observation, 23% of cases result in injury to one organ, 34% to two organs, and 9% to three organs (13, 14). Organ involvement may include the central nervous system (28%), cardiovascular system (25%), lungs (23%), and kidneys (50%) (15, 16). Cerebral palsy resulting from neonatal HIE may also lead to multiple organ dysfunction (17). Renal biomarkers of neonatal HIE, including low molecular weight proteins, such as b2-microglobulin, myoglobin, retinol binding protein, and N-acetyl-b-D-glucosaminidase (NAG), have been widely studied and used to detect renal tubular dysfunction (15, 18). Of these, b2-microglobulin and NAG have shown promise. However, the clinical application of these biomarkers is not widely available. A biomarker that can be measured by a simple urine analysis would therefore be a valuable tool for assessing neonatal HIE severity.

Common serum biomarkers include lactate (19, 20), bilirubin, liver function tests, l*actate dehydrogenase* (LDH), troponin-T, creatine phosphokinase (CK), urine ratio of lactate/creatinine (L/C), interleukin (IL)-10 (11, 21), and plasma osteopontin (OPN) and glial fibrillary astrocytic protein (GFAP) (22). Serum IL-6 levels and ratio of L/C ratio may predict the disability and mortality in neonatal HIE (23). The acylcarnitine profile, which demonstrates the value of butyrylcarnitine as a prognostic marker, has been used in neonatal HIE (24). Studies have exhibited that biomarkers such as brain-specific proteins (neuron-specific enolase [NSE], S100B, ubiquitin carboxy-terminal hydrolase-L1, and total Tau) and cytokines (tumor necrosis factor alpha, interferon-gamma, IL-6, IL-1β, IL-10, IL-13, and brain-derived neurotrophic factor) are helpful for diagnosing HIE and predicting neurodevelopmental outcomes (25–28).

The kidney is fundamentally associated with the injuries caused by neonatal HIE. Damage to the kidney causes renal injury, which can lead to oliguria or anuria, renal proteinuria, and hematuria, which could be useful for identifying perinatal asphyxia. Early elevation of microproteins in the urine may associated with the severity of neonatal HIE and help predict the outcomes of encephalopathy in newborns being discharged (15, 18). In asphyxiated neonates, early protein elevation in the urine may be a marker for predicting neonatal HIE; however, its relation to short-term and long-term outcomes has not been adequately studied. Additionally, while the urine L/C ratio might be a useful biomarker for differentiating newborns with moderate-to-severe HIE from those with mild HIE, however, urine creatinine analysis requires a large amount of urine. Using a simple urine analysis to predict the severity of neonatal HIE would therefore be a valuable tool.

Early diagnosis and initiation of hypothermia therapy for neonatal hypoxic–ischemic encephalopathy (HIE) are critical within the first 6 h after birth. Collecting urine, especially from neonates with HIE, can be challenging and time-consuming post-birth due to the likelihood of renal injury. We assessed whether urine protein levels, measured via a simple urinalysis on the first day, could correlate with the outcomes of neonatal HIE.

## 2 Patients and methods

### 2.1 Patients

Data on patients born between 2015 and 2021 at the Chung Shan Medical University Hospital (CSMU) with neonatal HIE were collected and reviewed, including the following: history of placental abruption, cord prolapse or fetal distress (a heart rate < 100 bpm before birth), metabolic acidosis (arterial pH of <7.20 or base deficit of ≥10 mmol per liter), or dependent positive-pressure ventilation immediately beyond the tenth minute of life after birth. Neonatal HIE was classified according to the clinical Sarnat staging as follows: stage I (mild), stage II (moderate), and stage III (severe) (7, 8, 29). The CSMU is a medical center in the middle of Taiwan. The initial urine sample was collected on the first day after admission for analysis. Serum lactate, LDH, and troponin levels were analyzed as biomarkers in the first 6 h of life. For patients with stage I–III HIE, the series examination included a head *ultrasound* (HUS), m*agnetic resonance imaging* (MRI), and electroencephalogram (EEG) monitoring, if available. To classify Sarnat staging before 6 h after birth, an experienced pediatric neurologist and neonatologist were consulted after admission to classify them into Sarnat stage I (mild), II (moderate), and III (severe) HIE (1).

### 2.2 Urine collection and interpretation

The urine analysis findings included the color, appearance, specific gravity, pH, protein, urine glucose, ketones, urine red blood cell (RBC), white blood cell (WBC), leukocyte esterase, nitrite, bilirubin (30), and urobilinogen. The urine samples were analyzed with either the Sysmex UC-3500 or UF-5000 (Sysmex cooperation, Kobe, Japan), was dropped onto each pad of a dedicated test strip within the UC-3500 analyzer. The entire sequence, from sample aspiration, to color comparison, and the final output of the results, is fully automatic. The test paper MEDITAPE UC-9A (Sysmex cooperation, Kobe, Japan) was used. UF-5000 fluorescence flow cytometry technology and hydrodynamic focusing was also applied for urine sediment analysis. Each patient’s first urine sample on the first day was sent for urine analysis and the data, including urine RBC count per high power field (HPF), WBC count per HPF, and urine protein level were analyzed. The patients were classified according to urine protein level into group 1 [zero or trace levels (0 to 30 mg/dL)], group 2 [“+” (30 to 100 mg/dL)], group 3 [“++” (100 to 300 mg/dL)], and group 4 [“+++” or more (> 300 mg/dL)].

### 2.3 Evaluation of the neurological outcomes in neonatal HIE

To assess the neurological outcomes of neonatal HIE, MRIs, hearing assessments, a history of seizures before the age of 1 year, and neurodevelopmental examinations of patients aged ≥1 year were evaluated. An unremarkable MRI indicated that there were no visible lesions in the brain parenchymal region; a mild abnormal MRI indicated brain lesions in the parenchyma but not the basal ganglion, thalamus, midbrain, or brainstem; and a severe abnormal MRI indicated brain lesions as diffuse white matter involvements or brain lesions in one of basal ganglia, thalamus, and brain stem with or without involvement of other brain regions. All the newborns’ hearing was assessed using the newborn hearing screening for automatic auditory brainstem evoked response (aABR). For those patients who failed the aABR twice, ABR, otoacoustic emission (OAE), and steady state evoked potential (SSEP) tests were performed (31). The degree of hearing loss was classified as normal (≤ 35 dB nHL) or abnormal (> 35 dB nHL) (32, 33). P*atient outcomes of* neurodevelopment were evaluated at the corrected age of 1 year either through clinical evaluation or the Bayley Scales of Infant and Toddler Development, Third Edition (Bayley-III). Patient charts were retrospectively reviewed in all cases.

### 2.4 Statistical analysis

Significant differences were using the independent *t*-test to compare the means of two independent groups or the chi-squared test to compare the categorical variables. The Pearson correlation coefficient was provided to measure the linear association between two variables and the value *r* = 1 indicates a perfect positive correlation; the value *r* = −1 means a perfect negative correlation. In the case of a nonparametric sample distribution, a Mann–Whitney U test was performed. The Spearman’s Rho (*r_s_*) calculator was used to measure the strength of an association between two variables in the non-parametric test. The one-way analysis of variance (ANOVA) test for independent variables was to compare the means of 3 or more independent samples. Significance was set at *p* < 0.05.

## 3 Results

### 3.1 Demographic and clinical cardiopulmonary presentation of 83 newborns with HIE

**Figure 1:**
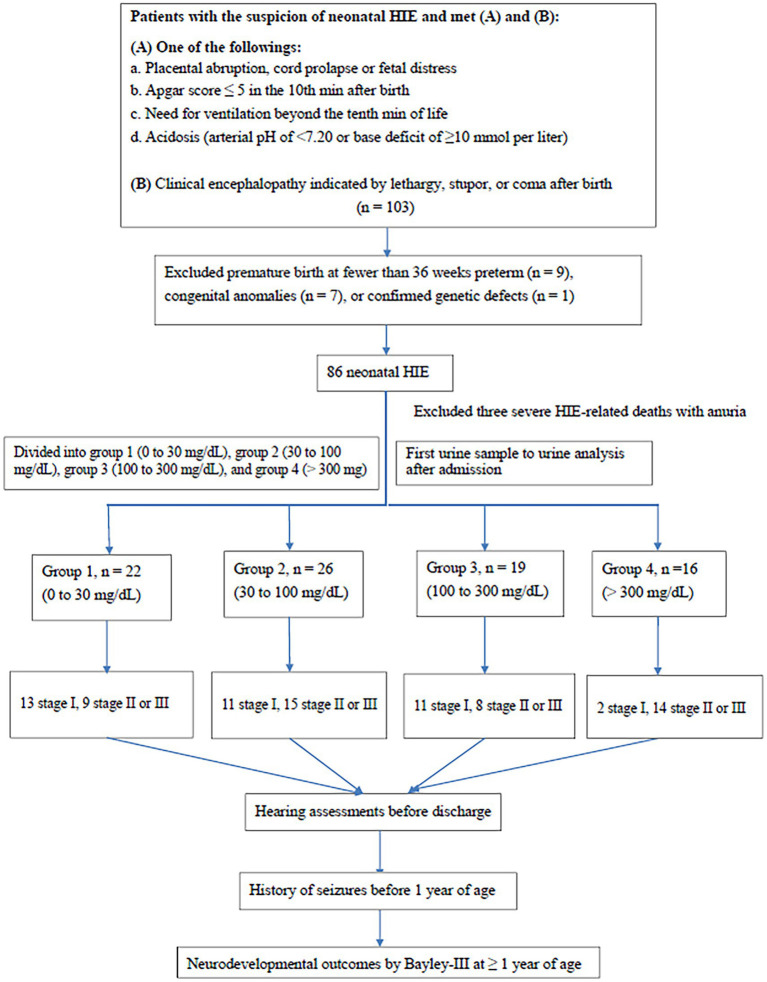
Flow chart of patient selection. MRI, magnetic resonance imaging. Bayley-III, Bayley Scales of Infant and Toddler development, third edition.

A total of 103 patients with HIE were enrolled. After excluding those with congenital anomalies (*n* = 7), premature birth (< 36 weeks; *n* = 9), and confirmed genetic defects (*n* = 1) (Figure 1), 86 cases were included. Three cases experienced post-birth mortality due to severe HIE and auria. Of the 83 cases, 37 had stage I (mild), 30 had stage II (moderate), and 16 had stage III (severe) HIE. The birth weight, gender, age, and method of delivery were not significantly different between the four groups according to urine protein level (Table 1). The time of first urine collection was 7.3 ± 3.5 h (range; 2–16 h) after birth in stage 1; 8.1 ± 3.7 h (range; 3–19 h) after birth in stage 2; and 8.7 ± 3.4 h (range; 3–13 h) after birth in stage 3. The timing of the initial urine collections were extended in advanced staging, but no significant differences were observed.

**Table 1:** Demographic data of 83 neonatal HIE cases according to urine protein level.

	Group 1^*^ (*n* = 22) 0 to 30 mg/dL8	Group 2^*^ (*n* = 26) 30 to 100 mg/dL	Group 3^*^ (*n* = 19) 100 to 300 mg/ dL	Group 4^*^ (*n* = 16) > 300 mg/ dL	*p values*
Gestational age (weeks), mean (SD)	38.4 (1.4)	38.1 (1.2)	38.4 (1.5)	38.1 (1.5)	f = 0.333, p = 0.802
Birth weight (gm), mean (SD)	2926.5 (173.6)	2975.1 (235.3)	2925.3 (216.0)	2906.3 (154.9)	*f* = 0.474, *p* = 0.701
Gender					
Male (*n* = 52) (100%)	12 (23.1%)	17 (32.7%)	10 (19.2%)	13 (25.0%)	*X2* (3, *n* = 83) = 3.88, *p* = 0.275
Female (*n* = 31) (100%)	10 (32.3%)	9 (29.0%)	9 (29.0%)	3 (9.7%)	
Delivery					
Inborn (*n* = 31) (100%)	9 (29.0%)	9 (29.0%)	7 (22.6%)	6 (19.4%)	*X*^2^ (2, *n* = 82) = 0.068, *p* = 0.966
Outborn (*n* = 52) (100%)	13 (25.0%)	17 (32.7%)	12 (23.1%)	10 (19.2%)	
Method of delivery					
Cesarean section (*n* = 39) (100%)	10 (25.6%)	12 (30.8%)	9 (23.1%)	8 (20.5%)	*X*^2^ (2, *n* = 82) = 0.040, *p* = 0.980
Vaginal delivery (*n* = 44) (100%)	12 (27.3%)	14 (31.8%)	10 (22.7%)	8 (18.2%)	

^*^Patients were divided into groups according to urine protein level as follows: group 1 (0 to 30 mg/ dL), group 2 (30 to 100 mg/dL), group 3 (100 to 300 mg/dL), and group 4 (> 300 mg).

### 3.2 Correlation between urine protein level and clinical stage

According to urine protein level, patients were divided into group 1 [zero or trace levels (0–30 mg/dL)], group 2 [“**+**” (30 to 100 mg/dL)], group 3 [“**++**” (100 to 300 mg/ dL)], and group 4 [“**+++**” or more (> 300 mg/ dL)]. Using *one*-*way ANOVA*, the urine protein level was significantly correlated with the staging (*f*-ratio 7.27, *p =* 0.007) (Table 2). Using linear regression analysis, ŷ (urine protein) = 0.26 (staging) + 1.40 (*r* = 0.36 according to the Pearson Correlation Coefficient Calculator) (*p* = 0.001, *n* = 83).

**Table 2:** Correlation between urine protein level and clinical staging, hearing impairment, history of seizures before 1 year of age, and neurodevelopmental outcomes at ≥1 year of age (*n* = 83).

	Group 1 (*n* = 22), 0 to 30 mg/dL	Group 2 (*n* = 26), 30 to 100 mg/dL	Group 3 (*n* = 19), 100 to 300 mg/ dL	Group 4 (*n* = 16), > 300 mg/ dL	*p* values
Clinical stagings					
Stage I (*n* = 37, 100%)	13 (35.2%)	11 (29.7%)	11 (29.7%)	2 (5.4%)	***X***^**2**^ **(3, *n* = 83) = 9.96, *p* = 0.019**^**$**^
Stage II and Stage III (*n* = 46, 100%)	9 (19.6%)	15 (32.6%)	8 (17.4%)	14 (30.4%)	
Neurodevelopmental outcomes ≥1 year (*n* = 83)					
Unremarkable in all (*n* = 53, 100%)	20 (37.7%)	17 (32.1%)	11 (20.8%)	5 (9.4%)	***X***^**2**^ **(3, *n* = 83) = 11.35, *p* = 0.009** ***X***^**2**^ **(1, *n* = 83) = 5.61, *p* = 0.018**^**+**^
Abnormal^*^ (*n* = 30, 100%)	4 (13.3%)	9 (30.0%)	6 (20.0%)	11 (36.7%)	
Hearing in all patients (*n* = 83)					
Normal (*n* = 73, 100%)	21 (28.8%)	24 (32.9%)	17 (23.3%)	11 (15.0%)	*X*^2^ (3, *n* = 83) = 1.952, *p* = 0.582
Abnormal (*n* = 10, 100%)	1 (10.0%)	2 (20.0%)	2 (20.0%)	5 (50.0%)	
History of seizures before 1 year of age					
Without seizure (*n* = 74, 100%)	21 (28.4%)	23 (31.1%)	17 (22.9%)	13 (17.6%)	*X*^2^ (3, *n* = 83) = 1.952, *p* = 0.582
With at least one seizure (*n* = 9, 100%)	1 (11.1%)	3 (33.3%)	2 (22.3%)	3 (33.3%)	

The bold font indicates *p* < 0.05; MRI, magnetic resonance imaging.

Patients were divided into groups according to urine protein level as follows: group 1 (0 to 30 mg/dL), group 2 (30 to 100 mg/dL), group 3 (100 to 300 mg/dL), and group 4 (> 300 mg).

^**$**^The odds ratio of stage II or stage III HIE when urine protein level was > 300 mg/ dL was 7.66 (*p* = 0.010; 95% CI, 1.61 to 36.33) compared with those patients with urine protein levels 0 to 300 mg/dL.

^*^Includes 2 cases that were discharged but died before 1 year of age.

^+^When groups 1 and 2 are compared with groups 3 and 4.

**Figure 2:**
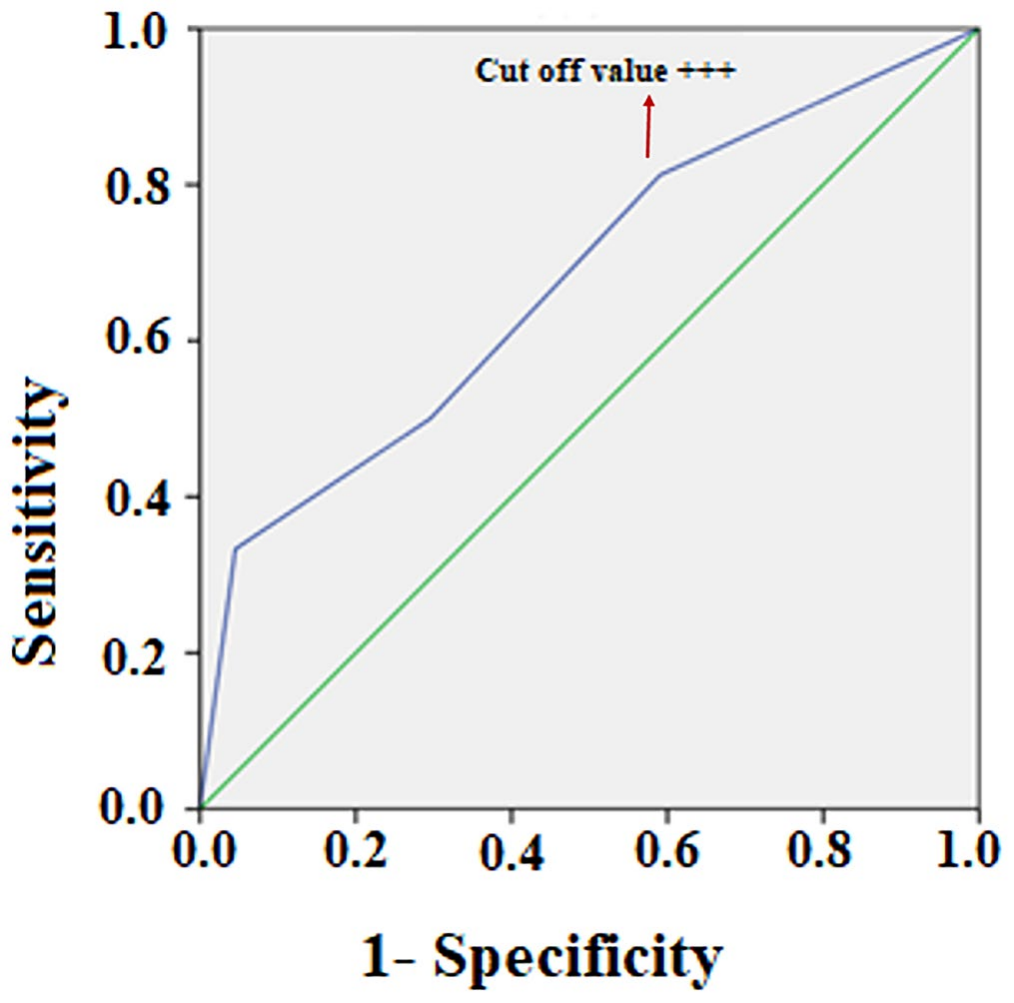
Receiver operating characteristic (ROC) curve of urine protein and cut-off value to predict HIE staging. ROC plots of urine protein and the area under the curve (AUC) for HIE (AUC 0.676, *p* = 0.004, 95% CI = 0.568–0.785). The cut-off value for maximizing AUC of urine protein was “+++” or more [> 300 mg/dL].

Table 2 shows the correlation between the HIE stage and urine protein levels. The urine protein classification among the four groups were significantly different [*X*^2^ (3, *n* = 83) **=** 9.96; *p* **=** 0.019]. Of the 48 neonates in group 1 and 2 (urine protein <100 mg/dL), 24 had stage I HIE (50%) and 24 (50%) had stage II or III; whereas of the 35 neonates in groups 3 and 4 (urine protein >100 mg/dL), 13 had the stage I (37.1%) and 22 (62.9%) had stage II or III HIE. Of the 16 neonates in group 4, only two (12.5%) had stage 1 HIE. The odds ratio of stage II or III requiring rescue hypothermia therapy for those with urine protein levels >300 mg/dLwas 7.66 (*p* = 0.010; 95% *confidence interval* (CI), 1.61–36.33) compared with those patients with urine protein levels <300 mg/dL. Receiver operating characteristic (ROC) curve of urine protein and cut-off value to predict HIE staging. ROC plots of urine protein and the area under the curve (AUC) for HIE (AUC 0.676, *p* = 0.004, 95% CI = 0.568–0.785). The cut-off value for maximizing AUC of urine protein was “+++” or more [> 300 mg/dL] (Figure 2).

A urine protein level ≥ 300 mg/dL had a high positive predictive value (87.50%; 95% CI, 0.63–0.97) and a high specificity (94.59%; 95% CI, 0.81–0.99) for stage II or stage III HIE requiring emergent hypothermia therapy, but had a low negative predictive value (52.24%; 95% CI, 0.47 to 0.57) and a low sensitivity (30.43%; 95% CI, 0.18–0.46) (Table 2).

### 3.3 Urine protein vs. lactic acid and LDH levels

Urine protein levels were significantly correlated with the first serum lactic acid level after admission according to linear regression analysis (*p* = 0.006; r(81) = 0.304; *n* = 83). However, urine protein levels were not significantly correlated with the first LDH level taken after admission according to the linear regression analysis [*p* = 0.198; *r*(81) = 0.304; *n* = 83].

### 3.4 Urine protein vs. creatinine level

Available urine protein levels was associated with the first serum creatinine; 0.84 ± 0.21 mg/dL in group 1, 0.94 ± 0.28 mg/dL in group 2, 0.97 ± 0.19 mg/dL in group 3, and 1.05 ± 0.21 mg/dL in group 4. The creatinine level increased with higher urine protein, and was significantly different in group 1 versus group 4 (*p* = 0.022).

### 3.5 Correlation between urine protein levels and MRI results, neurodevelopmental outcomes, hearing impairment, and seizures after discharge

**Figure 3:**
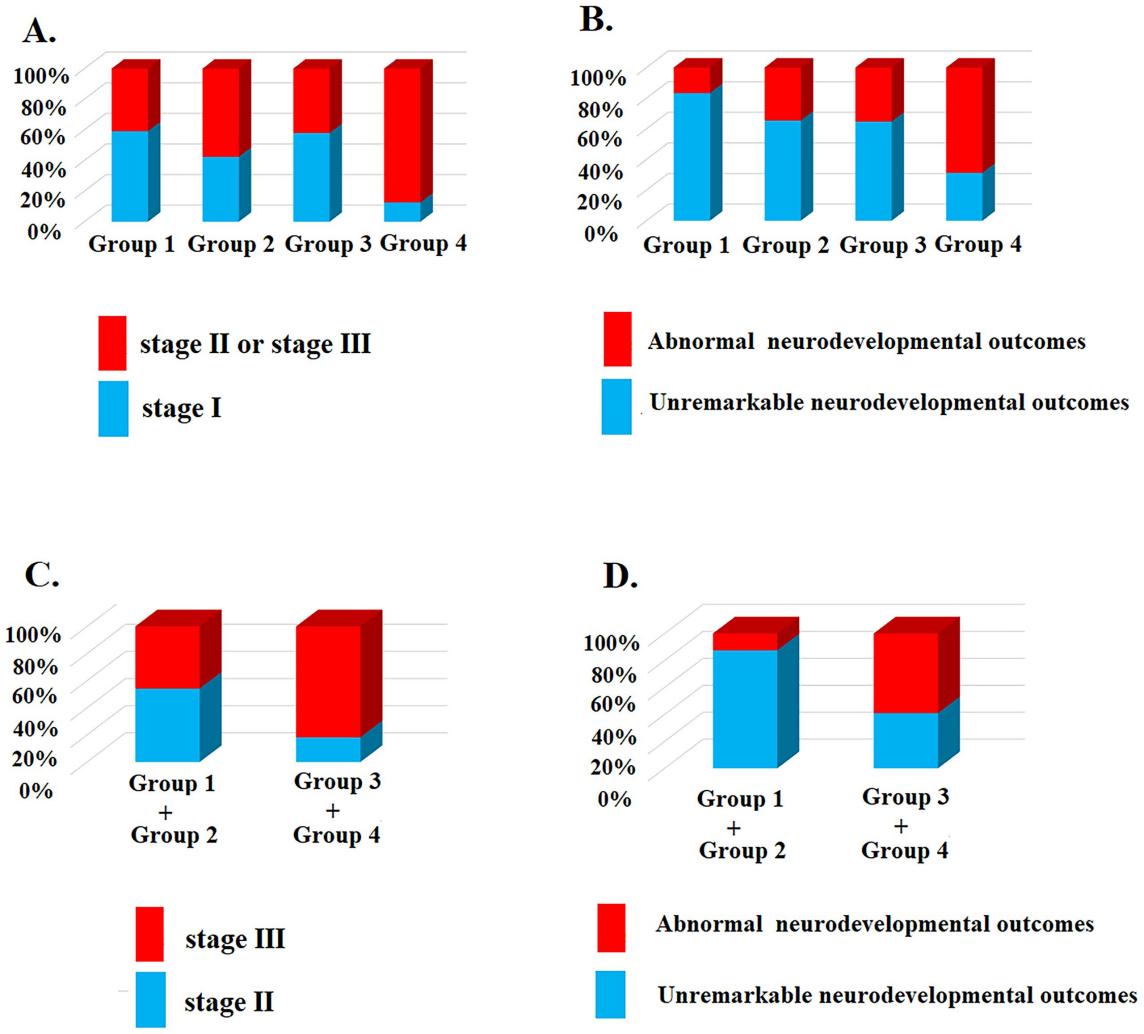
The urine protein level correlated with the clinical staging and neurodevelopmental outcomes ≥1 year old. **(A)** Patients were divided into groups according to urine protein level were significantly correlated with clinical staging [*X*^2^ (3, *n* = 83) = 9.96; *p* **=** 0.019]; **(B)** Urine protein levels were significantly correlated with neurodevelopmental outcomes at ≥1 year of age [*X*^2^ (3, *n* = 83) = 11.35; *p* = 0.009] for all HIE stages; **(C)** For those who underwent therapeutic hypothermia (clinical staging II and III, *n* = 46), more urine protein levels were significantly correlated with advanced clinical staging [*X^2^* (1, *n* = 46) =10.98; *p* < 0.001] and **(D)** neurodevelopmental outcomes at ≥1 year of age [*X^2^* (1, *n* = 46) = 6.38; *p* = 0.012].

Urine protein levels were significantly correlated with clinical staging [*X*^2^ (3, *n* = 83) = 9.96; *p* = 0.019] (Table 2 and Figure 3) and patient outcomes of neurodevelopment at ≥1 year of age [*X*^2^ (3, *n* = 83) = 11.35; *p* = 0.009] for all HIE stages, but not with the hearing outcomes or a history of seizures before 1 year of age (Table 2 and Figure 3). For those who underwent therapeutic hypothermia (*n* = 46), urine protein levels were significantly correlated with clinical staging [*X^2^* (1, *n* = 46) =10.98; *p* < 0.001] (Table 3 and Figure 3) and patient outcomes of neurodevelopment at ≥1 year of age [*X^2^* (1, *n* = 46) = 6.38; *p* = 0.012] (Table 3) but not with hearing outcomes, MRI finings, or a history of seizures before 1 year of age (Table 3 and Figure 3).

**Table 3:** Correlation between urine protein level and clinical staging, MRI findings, hearing impairment, history of seizures before 1 year of age, and neurodevelopmental outcomes at ≥1 year of age in patients who underwent hypothermia therapy (*n* = 46).

	Urine protein group 1 and 2^*^ at first day (*n* = 24, 100%)	Urine protein group 3 and 4^*^ at first day, (*n* = 22,100%)	*P* values
Clinical staging			
Stage II (*n* = 30)	21 (87.5%)	9 (40.9%)	***X***^***2***^ (1, *N* = 46) = 10.98, *p* < 0.001^**^
Stage III (*n* = 16)	3 (12.5%)	13 (59.1%)	
MRI finings^+^			
Unremarkable MRI (*n* = 23) in stage II and III	11 (45.8%)	12 (54.5%)	*X^2^* (1, *N* = 46) = 0.349, *p* = 0.555
Abnormal MRI	13 (54.2%)	10 (45.5%)	
Mild (*n* = 14)	9	5	
Severe (*n* = 9)	4	5	
Neurodevelopmental outcomes ≥1 year (*n* = 48)			
Unremarkable (*n* = 17)	13 (54.2%)	4 (18.2%)	***X***^***2***^ **(1, *n* = 46) = 6.38, *p* = 0.012**
Abnormal (*n* = 29)^#^	11 (45.8%)	18 (81.8%)	
Hearing in hypothermia group			
Normal (*n* = 38)	21 (87.5%)	15 (68.2%)	*X^2^* (1, *N* = 48) = 2.52, *p* = 0.113
Abnormal (*n* = 10)	3 (12.5%)	7 (31.8%)	
History of seizures before 1 year of age			
Without seizure (*n* = 37)	21 (87.5%)	16 (72.7%)	*X^2^* (1, *N* = 46) = 1.5917, *p* = 0.207
With at least one seizure (*n* = 9)	3 (12.5%)	6 (27.3%)	

The bold font indicates *p* < 0.05; ^**^p < 0.005. NE, neonatal encephalopathy; SD, standard deviation; MRI, magnetic resonance imaging.

^#Includes 2 cases who died before 1 year of age.^

^*^Patients were divided into groups according to urine protein level as follows: group 1 (0 to 30 mg/dL), group 2 (30 to 100 mg/dL), group 3 (100 to 300 mg/dL), and group 4 (> 300 mg).

^+^Unremarkable changes in brain parenchymal MRI; mild abnormal MRI indicates brain lesions in the parenchyma but not the basal ganglion, thalamus, midbrain, or brain; severe abnormal MRI indicated brain lesions as diffuse white matter involvements or brain lesions in one of basal ganglia, thalamus, and brain stem with or without involvement of other brain regions.

### 3.6 Urine RBC count (RBC/ HPF) vs. clinical staging and blood biomarkers for neonatal HIE

According to urine RBC count, patients were classified into group A (urine RBCs**/** HPF ≤ 5), group B (urine RBCs**/** HPF > 5 and ≤ 10), and group C (urine RBCs**/** HPF > 10). According to the linear regression analysis, none of the groups were significantly correlated with clinical staging (*p* = 0.728), LDH levels (*p* = 0.121), or lactic acid levels (*p* = 0.198). Thus, the urine RBC count was not correlated with clinical staging or blood biomarkers associated with neonatal HIE.

### 3.7 Urine WBC count (WBC/ HPF) vs. clinical staging, LDH, and lactic acid levels

According to urine WBC/ HPF count, patients were classified into the following groups: ≤ 5, > 5 and ≤ 10, and > 10 WBCs/ HPF. According to the linear regression analysis, the urine WBC count was not correlated with clinical staging (*p* = 0.519), LDH level (*p* = 0.934), or lactic acid level (*p* = 0.549). Thus, the urine WBC/ HPF was not correlated with clinical staging or blood biomarkers associated with neonatal HIE.

## 4 Discussion

This study makes significant contributions by delineating the first-day urine protein levels after birth and assessing their correlation with both the clinical Sarnat staging of neonatal HIE and patient neurodevelopmental outcomes at ≥1 year of age. Other outcomes, such as hearing impairment and a history of seizures before 1 year of age, were not associated with the urine protein level. Urine protein levels after birth serve as a biomarker to identify neonates with HIE. However, other urine biomarkers like RBC and WBC counts showed no association with clinical staging or neurodevelopmental outcomes.

*These findings reflect the fact that renal injury could be associated with the degree of brain injury in patients with neonatal HIE.* Neonatal HIE can cause multiple organ injury, including of the brain, liver, heart, and kidney. Neonatal HIE can cause renal injury, but cause less injury to brain could occur, which in some cases is reflected as severe urine proteinuria with less brain injury. *Urine collection, particularly in neonates with HIE, may be difficult after birth, as oliguria or anuria are potential symptoms* (15)*. Indeed, anuria could be a sign of severe HIE* (13, 15, 34)*. Patients* who underwent therapeutic hypothermia had less renal impairment than those who did not undergo hypothermia therapy. Kidney injury may persist in asphyxiated newborns despite improvement in serum creatinine (34). The positive predictive value of oliguria was moderate for neonatal HIE, though collecting urine to measure urine creatinine and other urine biomarkers may not be possible in this population (15). Urine protein levels were not associated with MRI findings in those who underwent therapeutic hypothermia, which probably was due to the fact that the treatment protected the brain from injury.

Blood troponin and LDH levels were not significantly correlated with urine protein levels in our study. This may be explained by the different degree of individual organ injury in patients with neonatal HIE (14). Lactic acid levels are more sensitively correlated with the degree of neonatal HIE than LDH and troponin levels (19, 20, 35). Early elevation of lactic acid levels may be correlated with the severity of neonatal HIE and help predict residual encephalopathy in newborns at discharge (20, 36). Troponin levels were found to be significantly higher in neonates with hypotension and injury of liver but not acute renal injury in neonatal HIE (5, 36).

This study had some limitations. First, a limited number of cases of HIE were included to analyze, which could have introduced bias into our findings. The data also had some bias related to HIE, which comprise fewer cases. Therefore, future studies that include a greater number of cases are warranted. However, the level of bias should be reduced since our patients were taken from a specific population without differences in gestational age, body weight, or gender, and those with medical conditions other than HIE (congenital anomalies, genetic disorder, or preterm birth) were excluded. Second, aggressive imaging studies were not available for those patients with stage 1 HIE and favorable outcomes. However, to confirm the absence of significant brain parenchymal lesions, patients in this population could undergo a series of HUS and clinical follow-up for 1 year to support the image finding. Third, urine analyses are often difficult to perform after birth as some patients have renal damage which may limit its clinical application, particularly for early hypothermia therapy. Collecting the first urine with a *Foley* catheter is an alternative choice for urine collection. Because urine creatinine analysis requires a large amount of urine, the creatinine values were not examined in HIE patients in our patients. However, measuring urine protein from a small sample could be utilized as a biomarker for predicting neurodevelopmental outcomes.

## 5 Conclusion

High levels of urine protein detected in a simple analysis of the first urine sample taken within the first day after birth may be associated with neurodevelopmental outcomes in patients aged one year or older. Using urine protein levels as a biomarker for neonatal HIE could serve as a tool to correlate with neurodevelopmental outcomes at ≥1 year of age.

## Data Availability

The original contributions presented in the study are included in the article/supplementary material, further inquiries can be directed to the corresponding author/s.
